# Delirium Tremens Successfully Treated by Chloroform

**Published:** 1853-01

**Authors:** Jonathan Letherman

**Affiliations:** Assistant Surgeon, U. S. Army; Fort Meade, Florida


*Delirium Tremens Successfully treated by Chloroform.* By JONATHAN LETHERMAN, M. D., Assistant Surgeon, U. S. Army.

Francis Harvey, a Hollander by birth, is now, and for a long period has been, in the service of the United States ; is forty-two years of age, weight one hundred and eighty pounds, is five feet seven and one-fourth inches in height, and measures thirty-eight inches around the chest.

This person had been absent from his Post for a few days in December, 1851, during which time he drank a large quantity of alcoholic liquors. This indulgence induced a severe attack of dysentery, which continued for five days after he reported sick on the 26th of December, and then entirely ceased.

Upon the subsidence of this disease he was seized with well marked symptoms of delirium tremens. The day following his recovery he was perfectly sane. During the previous night, however, he was slightly wandering in his mind, yet not to such a degree as to require sending for me. But during the night of January 1st, 1852, I was called up to see him, and, upon visiting the Hospital, found him trembling violently, having a great dread of injury, and a sensation as if a serpent were in his head and breast. He was fearful of every one who came nigh him, but when informed who I was his fear of me much abated. His pulse was frequent and weak, his eyes infected with contracted pupils, and his countenance exhibiting a vacant stare. A large dose of tincture of opium was ordered. The following morning, finding him nothing improved, I directed porter and laudanum at intervals throughout the day, and also the compound infusion of Cinchona.

During the week ending January 9th, this treatment was continued, except, that finding the failure of opium and stimulants in combination, these articles were ofttimes given separately, attention being given to the diet, which was generous, and to the organs of digestion. The patient, during this period, occasionally experienced a slight sleep, or, more properly, a stupor, after having taken a large opiate and stimulants, but it was so slight as to be of no service and totally unrefreshing. The trembling and fear of injury disappeared, but he was constantly annoyed by the idea (of the truth of which he seemed fully impressed) of animals in his head and breast, now of large beasts, now of serpents. Notwithstanding the subsidence of some of the symptoms, the patient at this time was no better. The tongue was coated and the pulse frequent and weak. Blue pill was directed, and Brandy ℥ij., Tinc. opii. gtt. 60, aq. ℥ij. was ordered every fifteen minutes until the patient should begin to experience sleep. This latter prescription was continued for seventeen hours, and although he was somewhat more tranquil, he yet had no sleep, and I could not pronounce him better. On January 10th the principal symptoms persisted, and I directed Brandy ℥ij., Tinc. opii. ʒij., aq. ℥ij. to be given every half hour until he should exhibit indications of sleeping. This was administered during the day, but produced not the desired effect. In other cases I had used opiates and stimulants in combination and with success.

From the 10th until the 16th of the month, the unpleasant symptoms would somewhat abate after the administration of large doses of laudanum, but, upon the withdrawal of the drug, or the diminution of the dose, he would relapse into his former condition. As yet he had no refreshing sleep. On the 16th, Porter ℥ij., Tinc. opii. ʒij., Chloroform gtt. xx. were directed every hour, to be discontinued should any indications of sleep appear. The day following he was a little better, but had not slept. The directions of the 16th were enforced, the laudanum excepted. On the 1st of February the patient had slightly improved, but yet had slept none. Whilst taking chloroform he was a little better, yet not sufficiently so to be satisfactory; had wandering pains in his head and breast. Ordered chloroform ʒi. in two ounces of water every 4th hour, porter ℥iij. every 2d hour. After the administration of the chloroform the pain in the head was relieved, the whole system rendered quiet and composed, and, for the first time during a period of five weeks, the patient slept well throughout the night. Very shortly after the first dose of chloroform was given emesis occurred, but a sinapism to the epigastrium relieved all disposition to vomit, and it happened no more.

Under this treatment the patient rapidly improved, and excepting an attack of intermittent fever, a disease which is rife in this country, nothing intervened to retard his recovery. On the 25th of February he was considered able to undergo the exposures incident to frontier service, and was consequently reported for duty.

He continued well until the first of March, when his desire for liquors, which at that time was uncontrollable, again led him astray. He was reported sick to me on the evening of March 3d, and I found him affected with delirium tremens, having the "feeling as if the devil were in his head," and headache. Directed three cups to nucha, and one drachm of chloroform, and left, with orders to be called should any untoward symptoms appear during the night. He felt much relieved after the application of the cups, and slept through the night. The succeeding morning he complained of his head, had a slight fever, no appetite, and a coated tongue; notwithstanding, he was better. Directed Tincture of Valerian half a drachm every 4th hour, and a drachm of Chloroform every 4th hour, commencing two hours after the Tinc. Valerian. The morning following the patient was perfectly sensible; bowels regular, appetite good, and had slept well throughout the night. The Tinc. Valerian was omitted and chloroform alone given in gradually diminishing doses until the 7th of that month, when he ceased to take any medicine, and was again returned to duty on the 15th. On the 21st he left the Post with his company and marched forty-seven miles to a station farther in the interior of the State.

This man has been in the habit, for some years, of occasionally indulging to excess in alcoholic liquors, and had he not been endowed with a vigorous constitution, would doubtless have long since paid the penalty of such excesses. His case interested me much by reason of its severity, its long duration, and the total failure of opiates and varied stimulants, administered separately and in combination, in affording relief. The failure of the preparations of opium was not to be attributed to a want of being given in quantities sufficiently large, for they were administered, not by the ordinary doses, but regulated according to their effects as much as possible.

The quantity of chloroform first exhibited was too small. At that time, and for some time afterwards, I was not aware that it had ever had been given in this disease, and was induced to direct it from the effects I had noticed in other diseases. It then failed in procuring sleep, yet it caused a perceptible improvement, and I then took into serious consideration the propriety of largely increasing the dose, and the probable effects thereof. At that juncture, I received the American Journal of Medical Sciences for the 4th quarter of 1851, wherein I was much pleased to see the record of a case in which it had been administered with profit in larger doses. I had no longer any lingering doubts, and gave it in such doses, and the result was gratifying in the extreme.

That it was the remedial in this case can scarcely be doubted, and should any doubts exist as to its efficacy iu the first attack, its influence in the second can hardly be questioned. The minor details of treatment have not been given, as the object has been to contribute additional testimony to the value of chloroform in this disease, in controlling which it has acted so speedily and so well.

Attention was given to the different organs of the body, and remedies from time to time were administered to keep them, so far as possible, in a healthy condition.

This man is now in the enjoyment of robust health, and has been so for many months.

Since I treated this case, I have used this drug in several cases of this disease, and, with one exception, it answered my expectations fully. It has brought relief, when opiates, instead of diminishing, added to the evil influences of the disease.

*Fort Meade, Florida*, Dec. 1st, 1852.

